# Fractionated photoimmunotherapy stimulates an anti-tumour immune response: an integrated mathematical and in vitro study

**DOI:** 10.1038/s41416-024-02844-y

**Published:** 2024-09-11

**Authors:** Mohammad U. Zahid, Matthew Waguespack, Rebecca C. Harman, Eric M. Kercher, Shubhankar Nath, Tayyaba Hasan, Imran Rizvi, Bryan Q. Spring, Heiko Enderling

**Affiliations:** 1 Department of Radiation Oncology, The University of Texas MD Anderson Cancer Center, Houston, TX USA; 2 Department of Physics, Northeastern University, Boston, MA USA; 3 Wellman Center for Photomedicine, Massachusetts General Hospital and Harvard Medical School, Boston, MA USA; 4 Joint Department of Biomedical Engineering, University of North Carolina at Chapel Hill and North Carolina State University, Chapel Hill, NC USA; 5 Lineberger Comprehensive Cancer Center, School of Medicine, University of North Carolina at Chapel Hill, Chapel Hill, NC USA; 6 Department of Bioengineering, Northeastern University, Boston, MA USA; 7 Institute for Data Science in Oncology, The University of Texas MD Anderson Cancer Center, Houston, TX USA

**Keywords:** Ovarian cancer, Computational science, Nonlinear dynamics

## Abstract

**Background:**

Advanced epithelial ovarian cancer (EOC) has high recurrence rates due to disseminated initial disease presentation. Cytotoxic phototherapies, such as photodynamic therapy (PDT) and photoimmunotherapy (PIT, cell-targeted PDT), have the potential to treat disseminated malignancies due to safe intraperitoneal delivery.

**Methods:**

We use in vitro measurements of EOC tumour cell and T cell responses to chemotherapy, PDT, and epidermal growth factor receptor targeted PIT as inputs to a mathematical model of non-linear tumour and immune effector cell interaction. The model outputs were used to calculate how photoimmunotherapy could be utilised for tumour control.

**Results:**

In vitro measurements of PIT dose responses revealed that although low light doses (<10 J/cm^2^) lead to limited tumour cell killing they also increased proliferation of anti-tumour immune effector cells. Model simulations demonstrated that breaking up a larger light dose into multiple lower dose fractions (vis-à-vis fractionated radiotherapy) could be utilised to effect tumour control via stimulation of an anti-tumour immune response.

**Conclusions:**

There is promise for applying fractionated PIT in the setting of EOC. However, recommending specific fractionated PIT dosimetry and timing will require appropriate model calibration on tumour-immune interaction data in human patients and subsequent validation of model predictions in prospective clinical trials.

## Background

Advanced and recurrent epithelial ovarian cancer (EOC) has poor outcomes, despite first-line treatments that result in greater than 75% disease remission [1, 2]. This may be due to the fact that advanced and recurrent EOC typically presents as disseminated peritoneal disease that may have significant occult micrometastases [1]. Even though immunotherapies have offered hope for controlling disseminated disease in other settings, immunotherapy has proven ineffective against EOC in clinical trials [3–7]. For example, a recent phase III trial combining immune checkpoint inhibitors and chemotherapy in EOC (JAVELIN Ovarian 200) was unsuccessful in improving progression free survival or overall survival [8]. This may have been due to an unfavourable tumour-immune microenvironment in EOC, which is typically considered immunologically cold in addition to the immune cytotoxicity of high-dose intensity chemotherapy.

A potential strategy to treat such disseminated disease is photodynamic therapy (PDT). PDT is a photochemistry-based therapeutic modality in which a non-toxic photosensitizer is locally injected or applied topically and then excited by red-near infra-red light in the 600–800 nm wavelength range to produce cytotoxic molecular species known as photodynamic action [9]. The anticancer action of PDT includes immunogenic cell death [10–17]. Bowel phototoxicity has proven limiting for safe PDT in the peritoneal cavity [18–20]. Cell-activatable antibody-photosensitizer conjugates, termed photoimmunotherapy (PIT), are selectively taken up by tumour cells to enable microscale tumour selectivity. EGFR-targeted PIT facilitates safe peritoneal PDT that spares normal tissue, including immune cells [18]. EGFR is an important molecule for targeting cancer cells and it is frequently overexpressed by human ovarian cancers [21, 22] and in many other carcinomas [23]. Additionally, it has been shown that low-irradiance and low-dose, non-ablative PDT promotes an anti-tumour immune response, whereas ablative dose regimens do not stimulate the immune system [24]. These observed effects for PDT may potentially be useful therapeutic approaches for manipulating the tumour-immune microenvironment and potentiating an anti-tumour immune response. However, the effects of these therapies are highly dependent on the design of the treatment regimen—including the photosensitizer dose, the irradiance (light dose-rate), and the total light dose—which makes the rational design of treatment regimens critical [24–27]. The low-dose, immune stimulating PDT regimens conserve molecular oxygen, induce inflammatory cytokines and neutrophil infiltration in the tumour required for and adaptive immune response [15]. The dose-dependent interactions of PIT with the immune system are less well-studied and motivate the present work.

To investigate the many possible combinations of treatment strategies, dosing and sequencing experimentally in both in vitro and in vivo settings and to decipher the complicated tumour-immune dynamics may be unfeasible [28]. Additionally, with such a non-linear dynamical system, simple linear extrapolations are likely insufficient to predict the immunological consequences of a cancer therapy that is meant to perturb this system. Mathematical oncology is an emerging field that aims to model the complex, heterogeneous, non-linear dynamics that underly cancer development and response to therapy [29, 30]. There has been much work in modelling cancer-immune interactions and how different cancer therapies perturb these dynamics [31–35]. These approaches may be useful in identifying how to best use phototherapy to elicit immune-mediated tumour control.

Here we integrate in vitro measurements of EOC tumour cell and T cell responses to chemotherapy and phototherapy (both PDT and PIT) into an ordinary differential equation model for tumour and immune effector cell interaction. This model is then used to explore how repeated administration of low light dose for PIT could be utilised to effect tumour control via cumulative stimulation of an anti-tumour immune response.

## Methods

### Cell lines

The human EOC cell lines NIH:Ovcar3 (Ovcar3) and the NIH:Ovcar5 (Ovcar5) were obtained from ATCC (HTB-161) and from Fox Chase Cancer Center (Philadelphia, PA) under a Material Transfer Agreement (MTA), respectively. Derivatives of Ovcar3 and Ovcar5 cells, mCherry–Ovcar3 and EGFP–Ovcar5, that stably express mCherry fluorescent protein and EGFP (enhanced green fluorescent protein), respectively, were created in this study to monitor 3D spheroid growth and PDT treatment response following a previously published protocol [36]. The DsRed-expressing mouse T cells from OT–1 mice (C57BL/6–TgTcra/Tcrb homozygous, 1100 Mjb/J) were a generous gift from Professor Mei Wu (Wellman Center for Photomedicine, Massachusetts General Hospital and Harvard Medical School, Boston), and isolated from splenocytes following a published protocol [37, 38]. The Jurkat–GFP cell line was purchased from Fisher Scientific. Cultures were discarded after 28 passages or less and new vials were thawed as needed. All cell lines used in this study tested negative for Mycoplasma contamination (MycoAlert mycoplasma detection kit, Lonza). The cell lines did not undergo authentication after receipt from the vendors or after isolation from splenocytes.

### In vitro measurements of PDT, PIT and chemotherapy response

**Fig. 1:**
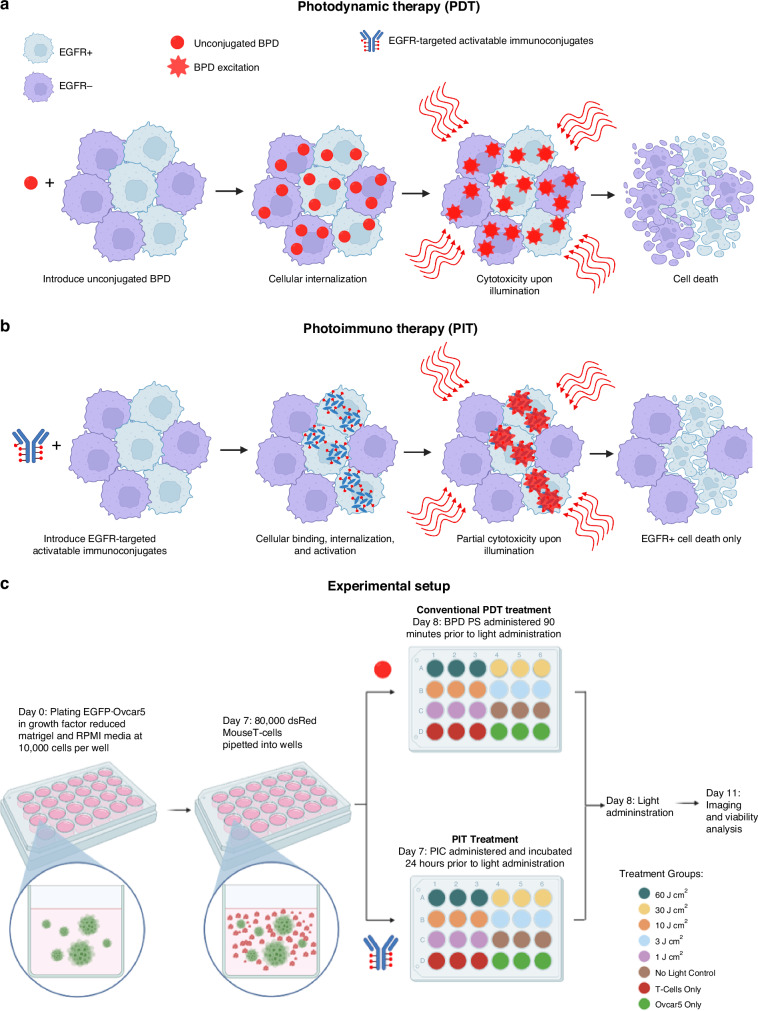
In vitro system for comparing photodynamic therapy (PDT) and photoimmunotherapy (PIT). Schematic depiction comparing the mechanisms of PDT (**a**) and PIT (**b**). In traditional PDT, unconjugated BPD is taken up indiscriminately by cells, resulting in cytotoxicity regardless of cell type. In PIT, EGFR-targeting immunoconjugates are selectively taken up by EGFR+ cells, resulting in selective killing of EGFR+ cancer cells. **c** Experimental timeline depicting treatment and control groups for PDT and PIT studies.

For PDT and PIT experiments, EGFP-Ovcar5 human cancer cells and dsRed expressing OT-1 mouse T cells were co-cultured in 3D using previously described methods [39]. This is a non-syngeneic model without a cognate antigen to facilitate specific T cell-mediated cytotoxicity. These were plated on to black walled, glass bottom 24 well-plates (Greiner Bio-One, 662892) for both PDT and PIT treatment arms. Conventional PDT used unconjugated benzoporphyrin derivative (BPD) given in 1 µM concentrations per well. PIT used cetuximab conjugated to BPD where concentrations were measured using BCA protein assay to provide a 1 µM equivalent BPD dose. Photosensitizer administration was followed by a 90 min (PDT) or 24 h (PIT) incubation time and a subsequent media refresh. A 690 nm diode laser at 150 mW/cm^2^ irradiance provided light activation of the photosensitizer. Energy doses were given at 60, 30, 10, 3, 1 and 0 J/cm^2^ with 3 biological replicates per group along with monoculture controls of each cell line (Fig. 1b). Here, a standard irradiance was used to test how PIT affects T cells at cytotoxic doses versus PDT and chemotherapy also administered at cytotoxic, lethal doses.

For chemotherapy, mCherry-Ovcar3 human cancer cells and EGFP-Jurkat human T cells were co-cultured in 3D. Jurkat cells are helper T cells that express CD4 but do not express CD8. Ovcar3 cells were used in this experiment to test a second EOC cell line and Jurkat T cells were used because the OT-T1 cell line was no longer available to us. Cisplatin doses of 300, 100, 30, 10, 3 and 1 µM were administered following the same plate pattern as the PDT and PIT plates of 3 replicates per dosage.

Ovcar3 and Ovcar5 cells express moderate levels of EGFR (27.9 and 111.1 normalised transcript expression values, nTPM, respectively; The Human Protein Atlas) compared to A431 (2978 nTPM, very high EGFR expression) and T47D (3.4 nTPM, low EGFR expression) cell lines.

After treatment each plate was incubated for 3 days and then imaged with an Operetta CLS high-content analysis system (Perkin Elmer, LIVE configuration) using LED excitation to collect z-stacks of both fluorescence channels and brightfield. A custom MATLAB code was used to reduce the images into a maximum intensity projection to produce a fluorescence-based histogram then following subtraction of the background signal the mean fluorescent protein fluorescence of corrected histograms were used to assess cell viabilities across each treatment modality.

### Calibrating dose response curves

Cell survival data for both tumour cells and T cells for each treatment were fit using least squares regression to the following equation,

1 $$S\left(d\right)={S}_{0}+\frac{{S}_{\infty }-{S}_{0}}{1+\exp ({m}_{{IC}50}{\log}\;d-\log (I{C}_{50}))}$$

where $S\left(d\right)$ is the fraction of surviving cells at dose $d$; ${S}_{0}$ is the untreated control survival fraction as treatment goes to zero; ${S}_{\infty }$ is the survival fraction as treatment goes to infinity; ${m}_{{IC}50}$ is the slope of the dose response curve at the $I{C}_{50}$ value; and $I{C}_{50}$ is the treatment dose at 50% of the maximum treatment efficacy.

### Mathematical model of cancer-immune dynamics

**Fig. 2:**
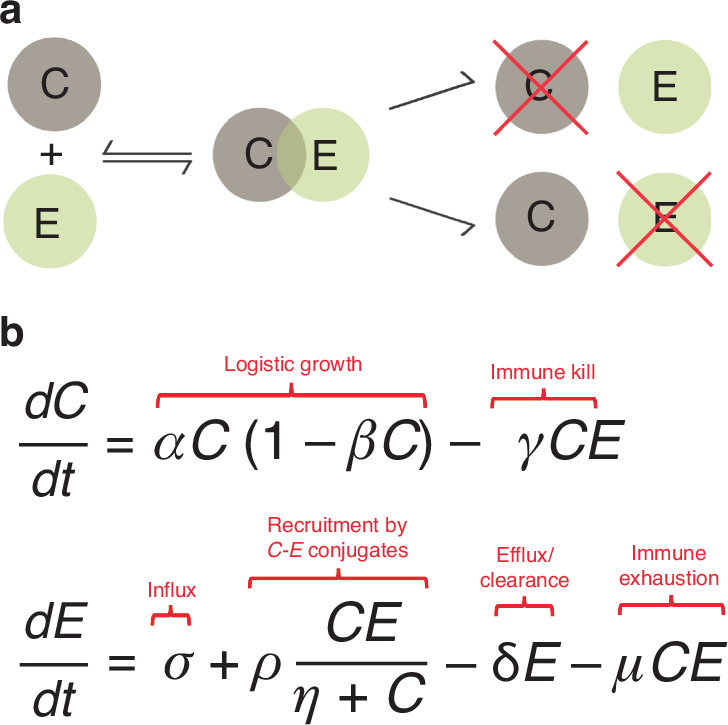
Mathematical model of tumour-immune effector dynamics. **a** Schematic depiction of tumour-immune effector cell interaction underpinning the model, which assumes that cancer (*C*) and immune effector (*E*) cells reversibly form a complex that can result in immune cell exhaustion or tumour cell death. **b** Coupled ODE system of the complete *C*-*E* dynamics, including immune influx, immune cell recruitment and clearance of immune cells, in addition to the interactions described above.

Our analysis is built on an established ordinary differential equation model of the interaction of tumour cells and immune effector cells, originally presented by Kuznetsov and colleagues [40]. This model assumes that when an immune effector cell (*E*) and a tumour cell (*C*) interact, then they form a complex, which can result in either the complex separating, via the reverse reaction, immune cell exhaustion, or tumour cell death (Fig. 2a). Additionally, the model assumes logistic tumour growth, a constant influx of *E* cells, autocatalytic recruitment of *E* cells by *C-E* conjugates in response to immunogenic cell death, and an exponential clearance of *E* cells. These dynamics are represented by a system of coupled ODEs shown in Fig. 2b. The non-dimensional unitless model parameters were set to the nearly the same values used in the original presentation of the model ($\alpha$=1.636, $\beta$=0.002, $\gamma$=1, $\sigma$=0.118, $\rho$=0.95, $\eta$=20.19, $\delta$=0.374, $\mu$=0.00311) [40]. The model was solved numerically in MATLAB, using the ODE solver *ode45*. The numerical solutions were evaluated with *E* over the interval of (0, 3.5) × 10^6^ cells at a resolution of 1.2 × 10^5^ cells, *C* over the interval of (0,450) × 10^6^ cells at a resolution 15.5 × 10^6^ cells, and a time resolution of 30 days per time step.

### Mapping dose response curves in the C-E phase plane

**Fig. 3:**
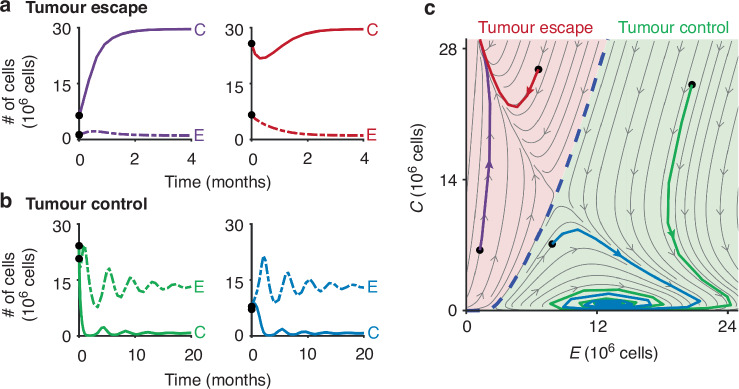
Dynamics of the cancer-effector model without treatment. **a** Example *C* and *E* time traces for two initial conditions (black circles) that result in tumour escape. **b** Example *C* and *E* time traces for two initial conditions (black circles) that result in immune-mediated tumour control. **c** The four initial conditions (black circles) and subsequent trajectories visualised in the *C*-*E* phase plane, demonstrating representative dynamics in the two basins of attraction (red: tumour escape; green: immune-mediated tumour control).

For demonstration purposes, dose response curves were generated in the phase plane (i.e. the mathematical analysis that plots the velocity of both time dependent variables against each other, c.f. Fig. 3c) by starting with initial values of *C*_0_ = 29 × 10^6^ cells and *E*_0_ = 4.1 × 10^6^ cells. However, later in the manuscript we will evaluate treatment response dynamics for all initial conditions (*C*_0_,*E*_0_) for which the untreated tumour escapes immune surveillance. Survival fraction (*S*) values were calculated for both *T* and *E* between 1 and 60 J/cm^2^ with a spacing of 1 J/cm^2^ for PDT (Verteporfin + light) and PIT (Verteporfin-Cetuximab + light) and between 1 and 100 μΜ with a spacing of 1 μM for chemotherapy (cisplatin). These SF values were applied to the initial *C* and *E* values to calculate the post-treatment cell numbers, which were then mapped onto the phase plane.

### Simulating fractionated photoimmunotherapy (PIT)

The initial fractionated PIT simulations started with the initial conditions of *C*_0_ = 29 × 10^6^ cells and *E*_0_ = 4.1 × 10^6^ cells. For each treatment schedule, first a *S*(d) for the given dose fraction was used to calculate the post-treatment (*C*, *E*) values, which are implemented as instantaneous changes in *C* and *E* numbers because of separation of timescales between the treatment effects (days-scale) and *C*-*E* population-level dynamics (months-scale). Next, the ODE system was solved forward in time for two simulation time steps (equivalent to 60 days). At this point, either the (*C*, *E*) value at this time point were set to the new initial conditions and the process was repeated for the remaining treatment fractions, or if this was the final treatment fraction in the schedule, then the ODE was solved forward until the system reached an equilibrium state.

The minimum number of PIT fractions required for tumour control was calculated for a given dose/fraction value (e.g. 1, 3, 5, or 7 J/cm^2^). This was done by simulating fractionated PIT, as described above and testing an increasing number of fractions until the minimum number yielding tumour control was found. This analysis was performed for every (*C*, *E*) initial condition to the left of the separatrix.

## Results

### Tumour-effector dynamics without treatment

The ODE system (Fig. 2b) results in two non-trivial equilibrium outcomes (1) tumour escape or (2) immune-mediate tumour control. In the case of tumour escape, immune effector cells are outgrown by the tumour cells, which grow to their equilibrium carrying capacity (Fig. 3a). In the case of immune-mediate tumour control, *C* and *E* cells undergo predator-prey oscillations [41, 42] resulting in an equilibrium state at low *C* numbers (Fig. 3b). We can visualise these interactions for all possible initial conditions (*C*_0_, *E*_0_) in the *C*-*E* phase plane (Fig. 3c), which visualises the two basins of attraction for the two outcomes described above.

### Treatment response evaluation

**Fig. 4:**
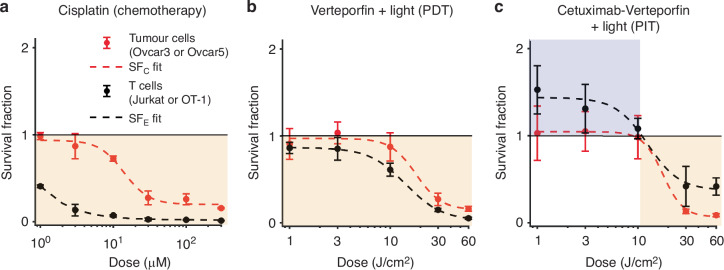
Cell survival data for chemotherapy and phototherapy. Dose response curves for tumour cells and T cells for chemotherapy (**a**), photodynamic therapy (**b**) and photoimmunotherapy (**b**) from *n* = 3 biological replicates for each treatment and cell type combination. Fits represent least-squares best fit to Eq. 1. Yellow background indicates regions where survival fraction (SF) < 1, tumour cell or T cell death; blue background where SF > 1, immune stimulation. All error bars indicate s.e.m. Note that the measurements in (**b**) and (**c**) were published previously [39]. Here, the data were re-analysed and normalised to the response to 1 J/cm^2^ to identify dose regimes of immune stimulation and cytotoxicity.

Chemotherapy resulted in lower T cell survival compared to tumour cell survival for all doses (Fig. 4a). Consequently, for chemotherapy to be successful in this simple in vitro model, treatment must eradicate every last cancer cell and cannot harness support from the immune system. Similarly, PDT resulted in lower T cell survival relative to tumour cell survival at higher doses, but the relative T cell survival fractions were comparable to the tumour cell survival fractions for lower light doses (Fig. 4b). On the other hand, PIT showed two distinct dose response regimes: (1) at low light doses (*d* ≤ 10 J/cm^2^) there is no discernible effect on tumour cells but the number of T cells significantly increased compared to untreated control; and (2) at high light doses (*d* > 10 J/cm^2^) both tumour cells and T cells experience cytotoxicity with survival fractions <1 (Fig. 4c). However, even at high doses, T cell survival fractions are greater than the tumour cell survival fractions, which is the opposite of what is seen for the other two treatment modalities.

### Simulating impact of treatments on tumour-effector state

**Fig. 5:**
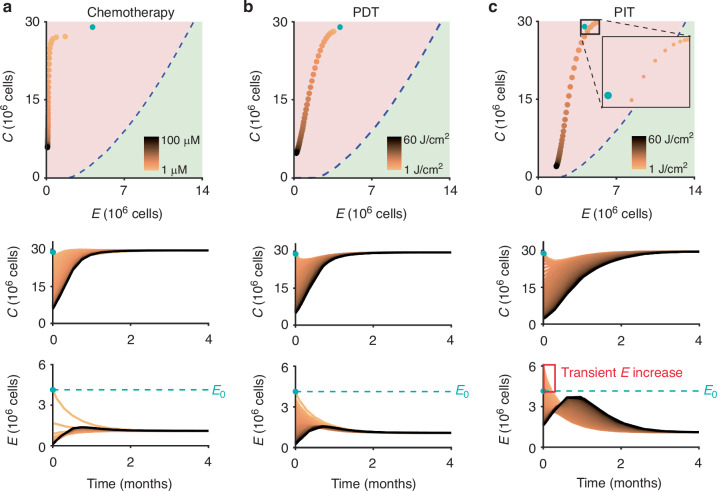
Dose response curves mapped onto the cancer-immune effector phase plane. The effect of each treatment (**a** chemotherapy; **b** photodynamic therapy; **c** photoimmunotherapy) were simulated for an example initial condition of *C*_0_ = 29 × 10^6^ cells and *E*_0_ = 4.1 × 10^6^ (blue circle) over the entire dose range (1–100 μM for chemotherapy; 1–60 J/cm^2^ for PDT and PIT) with the post-treatment cell numbers shown with copper-coloured circles on the *C-E* phase plane. Corresponding post-treatment time traces for the entire dose range for both cancer and immune effector cell populations are plotted below (colour scheme of individual trajectories corresponding to the treatment dose as in panels above).

We bring together our model and the dose-response data by mapping the dose responses of the different treatments onto the tumour-effector phase plane. This mapping demonstrates the effect of each treatment on the tumour-effector state over the entire dose range for a given initial set of initial conditions. Chemotherapy shows large reduction in effector cell number at the lowest doses with tumour cell number reduction only occurring with higher dose (Fig. 5a). In contrast, with PDT we see comparable reductions in tumour and effector cell numbers with increasing light dose, with greatest marginal reduction towards the middle of the dose range (Fig. 5b). Finally for PIT, there are two distinct behaviours: (1) in the *d* < 10 J/cm^2^ regime effector cell number increases with minimal reduction in tumour cell number and (2) in comparison to PDT and chemo, there is a decreased leftward shift (towards low effector cell numbers) as tumour cell number decreases with increasing dose (Fig. 5c). It should be noted that for the specific set of initial conditions shown here, all simulated treatments resulted in tumour escape over the entire dose range.

### Simulating fractionated photoimmunotherapy

**Fig. 6:**
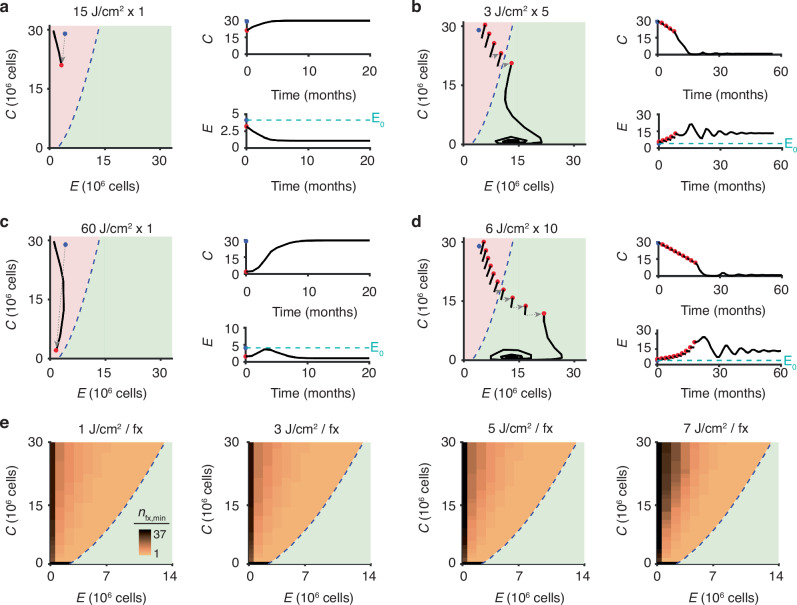
Simulations of fractionated photoimmunotherapy. Comparison of simulated PIT with a fixed total light dose (**a**, **b**: 15 J/cm^2^; **c**, **d**: 60 J/cm^2^) delivered in 1 fraction, resulting in tumour escape (**a**, **c**), versus delivery in multiple fractions of smaller dose, resulting in tumour control due to immune stimulation from low dose PIT (**b**, **d**). On both the phase planes and the *C* and *E* time trace plots, the blue circles indicate the pre-treatment initial conditions; red circles the (*C*, *E*) values after a PIT fraction; black curves the tumour-effector trajectories after a PIT fraction. **e** Simulated minimum number of PIT fractions (*n*_fx,min_) needed to achieve tumour control for a fixed dose/fraction size for all initial conditions left of the separatrix displayed as a heat map on the *C-E* phase plane.

In order to exploit the observed immunostimulatory effect of low-dose PIT, we simulated the novel approach of delivering a large total light dose that might otherwise not control a tumour in smaller dose fractions. The simulations demonstrate that while a single dose of 15 J/cm^2^ of PIT will yield an initial drop in tumour cell number, the tumour will eventually escape due to the unfavourable tumour-effector dynamics for the post-treatment (*C*, *E*) conditions (Fig. 6a). However, if this same total PIT dose is delivered in 5 fractions of 3 J/cm^2^, then the low-dose PIT will increase effector cell number with each fraction, eventually crossing the separatrix into a region of the state space that results in immune-mediate tumour control (Fig. 6b). The inter-fraction tumour cell number reductions are not a direct result of the preceding PIT dose, but rather due to the local tumour-effector dynamics.

There are similar results with a light dose of 60 J/cm^2^. When this dose is delivered in 1 fraction of 60 J/cm^2^, although there is a large initial drop in the tumour cell number, eventually the tumour escapes the effector cells (Fig. 6c). When the 60 J/cm^2^ dose is delivered in 10 fractions of 6 J/cm^2^, each fraction results in increased effector cell number, eventually leading to immune-mediated tumour control (Fig. 6d).

We tested the minimum number of PIT fractions required to achieve tumour control (*n*_fx,min_) for all (*C*, *E*) initial conditions to the left of the separatrix, across a range of fraction dose sizes. For all fraction dose sizes, initial conditions close to the separatrix needed a small *n*_fx,min_ to achieve tumour control (Fig. 6e). Initial conditions further from this boundary require larger *n*_fx,min_, and this number increases with increasing fraction dose size, as the immune stimulation decreases with larger dose (Fig. 4b).

## Discussion

We have demonstrated that by leveraging the immunostimulatory properties of the low-light dose regime of PIT, it may be possible to elicit immune-mediated tumour control in EOC using multiple administrations of low-light dose PIT. Here, the 3D co-culture models do not explicitly include specific T cell-mediated cytotoxicity through a cognate antigen, however, bystander killing by T cells is possible in these models and could conceivably contribute to PIT-mediated cell death. Note that it is conceivable that the transfected fluorescent proteins (EGFP and mCherry) used in these experiments could be exposed to immune cells following PDT or PIT, and these foreign proteins could help to elicit immune responses. Our modelling results suggest that even though a single larger dose of light for PIT will not shift the disease out of the tumour escape regime, fractionating the same total light dose into multiple administrations may enable tumour control without increasing the total light dose. Although repeated administration of PDT has been tested in pilot trials for glioblastoma [43] and skin cancer [44–46], to the best of our knowledge there has been no exploration of fractionated PIT.

It is important to emphasise that the motivation for fractionated (i.e., intermittent, low dose) PIT here is distinct from prior elegant research exploring fractionated dose regimens for PDT. Prior literature has explored fractionation in PDT as a means to improve efficacy by pausing irradiation to enabling reoxygenation of the tissue, thereby maximising the consumption of molecular oxygen and reactive oxygen species during the treatment session [47–55]. Here, in contrast, the focus is on immune stimulation and we do not model oxygen consumption. Given that low-irradiance, oxygen-conserving PDT regimens stimulate an adaptive anti-tumour immune response, future work could investigate similar dose regimens for PIT to help optimise outcomes building on elegant models of reactive oxygen species production and tissue molecular oxygen consumption [56, 57].

These results show promise for applying fractionated PIT in the setting of advanced and recurrent EOC where the disease is multifocal and spread throughout the peritoneal cavity and it is difficult to disentangle tumour from normal tissue. However, despite experimental data to calibrate treatment response, the underlying tumour-immune interaction model was calibrated on a different murine experimental setup and not EOC and immune cells in the treatment response experiments. Therefore, recommending specific dosimetry and timing for this treatment strategy will require appropriate model calibration and subsequent validation both in vitro and in vivo. Furthermore, in the presented mathematical model the effect of the immune system only includes immune effector cells. Future developments of this research must account for both the influence of and the effect of PIT on different immune cell types, especially immunosuppressive cell types, which will increase both biological and mathematical model complexity. It may be possible to do this by adding a third equation to the ODE system for immune suppressor cells. However, this will require the collection of appropriate in vitro data for immune suppressor cells for careful calibration of the new aspects of this future mathematical model.

The observation of T cell stimulation following low dose PIT (increased T cell fluorescent protein signal) above the no treatment control is a novel observation to the best of our knowledge. Future studies will test the mechanisms of T cell stimulation observed here for low dose PIT. There are reports of low-level light stimulation of cellular proliferation through endogenous chromophores, also called biomodulation [58] and activation of cell stress response activity to sub-lethal PDT [59]. Future work will also explore the role of T cell-mediated cytotoxicity in these in vitro models, and in immunocompetent in vivo syngeneic models and its interplay with PIT.

As mentioned already, EGFR is a promising target for PIT due to frequent overexpression in ovarian cancers [21, 22]. Further, EGFR-targeted PIT using IRDye700-cetuximab conjugates is in phase III clinical trials for recurrent head and neck cancer patients (NCT03769506, ASP-1929), with early conditional approval in Japan. EGFR monotherapy’s limited efficacy, hindered by various escape mechanisms [60], necessitates combination therapies. Our study focuses on PIT, which synergises photodynamic sensitisation with anti-EGFR agents [61–63]. PDT and PIT induce significant cell death and survival signalling (e.g. EGFR activation, VEGF secretion [64–66], priming cancer cells for concurrent molecular-targeted therapy (e.g. cetuximab), resulting in synergistic tumour reductions [59, 61, 67]. However, it is unlikely that a single antigen target or therapy will be sufficient to clear advanced and heterogenous EOC and future work is needed to expand the biomarker targets for PIT towards enable the targeting of multiple tumour biomarkers simultaneously depending on patient-specific tumour expression profiles.

If successful, many scenarios in which immune stimulation from fractionated PIT may be applied in cancer treatment are conceivable [68]. This could range from ‘mopping up’ residual disease post-surgical resection to priming an immunologically cold tumour for treatment with immune checkpoint inhibitors [69]. The delivery of light at multiple times for fractionated therapy will be a challenge for ovarian cancer and other internal malignancies. It may be possible to overcome this hurdle by using novel modes of low-irradiance and long-duration light delivery—e.g. implantable wireless devices [70] or non-invasive knitted light-emitting fabrics that can be wrapped around the abdomen [71]. Such an approach would take advantage of the fact that PIT is amenable to diffuse illumination due to the tumour selectivity of the photosensitizer delivery.

## Data Availability

The datasets generated during and/or analysed during the current study are available from the corresponding author upon reasonable request.

## Code availability

All code for data fitting, simulations and figure generation are available at the following GitHub repository: https://github.com/ZahidMU/FractionatedPIT_BritJCancer.
